# Neuroblastoma Presenting as a Congenital Renal Mass in a Neonate: A Diagnostic Pitfall

**DOI:** 10.3390/children13020283

**Published:** 2026-02-19

**Authors:** Agnieszka Sokół, Alicja Romaniak, Iwona Dachowska-Kałwak, Anna Wojtyłko, Marzena Kozakiewicz, Jan Godziński, Marek Ussowicz

**Affiliations:** 1 Department of Pediatric Bone Marrow Transplantation, Oncology, and Hematology, University Clinical Hospital, 50-556 Wroclaw, Poland; 2 Department of Pediatric Traumatology and Emergency Medicine, Wroclaw Medical University, 54-049 Wroclaw, Poland; 3 Department of Pediatric Surgery, Technical University of Wroclaw, 54-049 Wroclaw, Poland; 4 Department of Pediatric Bone Marrow Transplantation, Oncology, and Hematology, Wroclaw Medical University, 50-556 Wroclaw, Poland

**Keywords:** neonatal tumor, congenital renal mass, Wilms tumor, segmental chromosomal aberrations

## Abstract

**Highlights:**

**What are the main findings?**

- Neuroblastoma should be included in the differential diagnosis of a renal mass, particularly when the lesion involves the upper pole near the adrenal gland, even though congenital mesoblastic nephroma and Wilms tumor are statistically more likely in neonates.

**What are the implications of the main findings?**

- In very young infants, imaging findings can be challenging to interpret; radiological impressions should be considered provisional.
- Segmental chromosomal aberrations may predict poor prognosis even in apparently localized infant disease.

**Abstract:**

Background: Congenital renal masses in neonates are most commonly congenital mesoblastic nephroma or, less frequently, or Wilms tumor. We describe a neonate with an apparent primary renal tumor that proved to be adrenal neuroblastoma infiltrating the kidney, highlighting diagnostic pitfalls in this subgroup of patients. Methods: We retrospectively reviewed the diagnostic work-up, histopathology, genomic profiling, treatment, and outcome of a term neonate in whom a renal mass was detected incidentally on ultrasound. Results: Ultrasound and MRI showed a 2 cm solid lesion centered in the upper pole of the left kidney, interpreted as nephroblastomatosis/early Wilms tumor. Left nephrectomy with adrenalectomy revealed stroma-poor, undifferentiated neuroblastoma with regional node involvement and multiple segmental chromosomal aberrations, including 1p and 3p loss, but no MYCN or ALK alterations. Initial management consisted of surgery alone with close surveillance. Within weeks, early disseminated relapse with bone and soft-tissue metastases occurred, necessitating escalation to high-risk, COJEC-based chemotherapy; resection of residual mass; and modified consolidation without high-dose chemotherapy or radiotherapy. The child remains in complete remission with preserved renal function. Conclusions: Neuroblastoma should be considered in the differential diagnosis of congenital “renal” masses. Imaging-driven provisional diagnoses may be misleading, and genomic risk profiling may help lower the threshold for systemic therapy in selected cases.

## 1. Introduction

Pediatric renal tumors account for roughly 5–7% of childhood cancers, with Wilms tumor (nephroblastoma) representing about 80–90% of renal neoplasms in children. Wilms tumor typically presents between 2 and 5 years of age, and true neonatal Wilms tumor is rare [1]. In fetuses and neonates, the renal tumor spectrum is different [2]. Congenital mesoblastic nephroma (CMN) is the most common renal tumor in this age group and may account for the majority of renal tumors in infants younger than six months [3,4]. Malignant rhabdoid tumor of the kidney and very early-onset Wilms tumor are much less frequent but important to consider because of their aggressive behavior [5,6,7].

We report a neonate in whom an adrenal neuroblastoma infiltrating the kidney was initially interpreted as a primary renal mass (nephroblastomatosis/early Wilms tumor). The case illustrates how imaging-based clinical decision-making can be challenging in non-typical cases.

## 2. Materials and Methods

**Figure 1:**
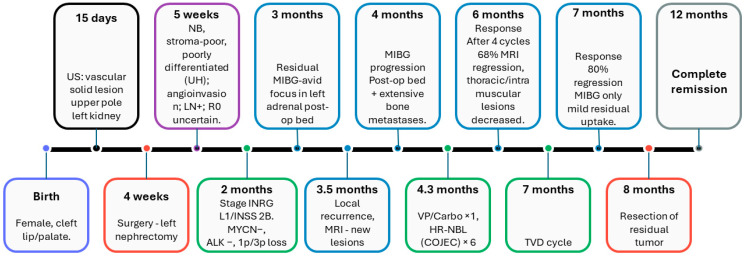
Clinical timeline of diagnosis, treatment, and follow-up.

We retrospectively reviewed the diagnostic work-up, histopathology, genomic profiling, treatment, and outcome of a term neonate in whom a renal mass was detected incidentally on ultrasound (Figure 1).

## 3. Results

A female infant was born at term (39 weeks) by spontaneous vaginal delivery, Apgar 10/10. Pregnancy was complicated by maternal hypertension and thrombophilia. Antenatal ultrasound demonstrated a cleft lip and palate; postnatal genetic testing on peripheral blood showed a normal karyotype. After birth, a left cleft lip and cleft palate (hard and soft) were confirmed. Postnatal adaptation was uneventful. The infant was clinically well, feeding normally, and gaining weight. There was no vomiting, abdominal distension, hematuria, hypertension, or fever. The abdomen was soft and non-tender without palpable masses or organomegaly. At approximately 2 weeks of age, during routine evaluation in a tertiary neonatal unit, a screening abdominal ultrasound was performed. Ultrasound demonstrated a small solid lesion in the upper pole of the left kidney. The child was referred to a pediatric oncology center for further assessment. Baseline blood tests, including full blood count and renal and liver function, were within normal limits for age. Pre-operative biochemical testing showed serum LDH 400 U/L (normal range 180–430). Ferritin and urinary catecholamine metabolites (VMA/HVA) were not obtained before surgery, as imaging favored nephroblastomatosis/early Wilms tumor, and the lesion was managed as a presumed primary renal tumor.

### 3.1. Imaging Studies

**Figure 2:**
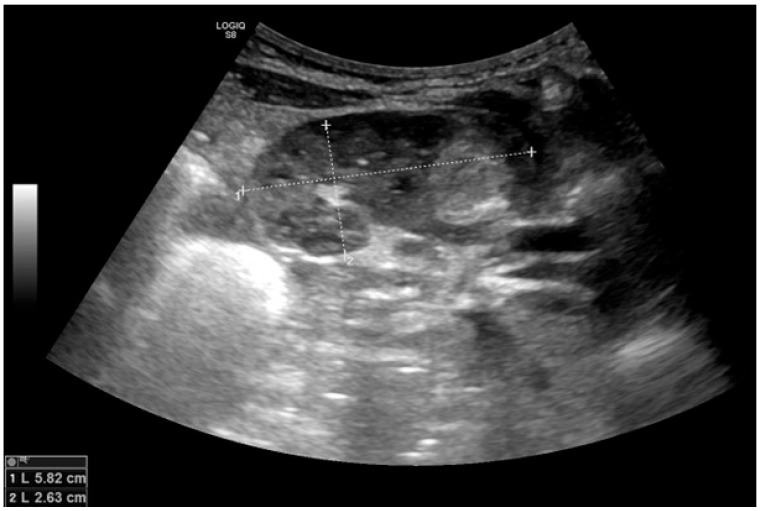
Abdominal ultrasound at diagnosis. Longitudinal sonographic view of the left kidney shows a well-defined, predominantly solid, mildly heterogeneous mass in the upper pole (calipers).

The repeated abdominal ultrasound confirmed a well-demarcated, moderately hyperechoic solid mass of approximately 2 cm in diameter, apparently centered in the upper pole of the left kidney, with mild dilatation of the upper calyceal system (Figure 2).

**Figure 3:**
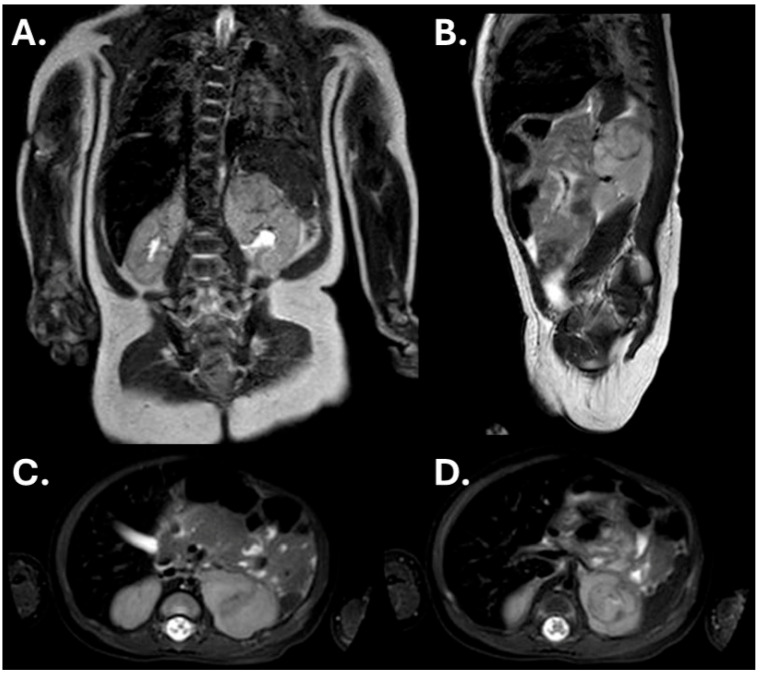
MRI of the abdomen at diagnosis. Coronal (**A**) and sagittal (**B**) T2-weighted images demonstrate a well-circumscribed solid mass centered in the upper pole of the left kidney. Axial T2-weighted fat-suppressed images (**C**,**D**) show a predominantly homogeneous solid tumor.

The right kidney and liver were normal; no lymphadenopathy was seen. The adrenal glands were reported as normal. Contrast-enhanced MRI of the abdomen showed a 2.0 cm solid lesion centered in the upper pole of the left kidney with relatively homogeneous signal, no gross necrosis or hemorrhage, no visible renal vein thrombus, and no encasement of major vessels (Figure 3).

The radiologist favored nephroblastomatosis or early nephroblastoma. Staging MRI of the brain and spine and CT of the chest, performed around the time of surgery, showed no intracranial, intraspinal, or pulmonary metastases.

### 3.2. Histopathology and Genomic Profiling

At 4 weeks of age, the patient underwent left nephrectomy with adrenalectomy and regional lymph node sampling via a left Chevron incision. Intra-operatively, the tumor was seen in the adrenal region with infiltration of the upper pole of the kidney, and the adrenal–renal interface was indistinct. The resection appeared macroscopically complete. For regional staging, locoregional lymph node sampling was performed from the left renal hilum/perirenal region.

Histopathology revealed a malignant tumor involving the adrenal gland and infiltrating adjacent renal parenchyma. Microscopically, it consisted of small, round, undifferentiated cells with scant cytoplasm and hyperchromatic nuclei, arranged in nests and sheets along with scanty neuropil, consistent with neuroblastoma, stroma-poor, undifferentiated type with low mitosis–karyorrhexis index.

Regarding nodal evaluation, one small hilar lymph node was negative for metastatic tumor. Separately, neuroblastoma tissue was found within perirenal adipose tissue, and the pathology report noted a differential of a satellite tumor focus versus metastasis within a lymph node (in the absence of clearly documented nodal architecture in that fragment). In the multidisciplinary review, this finding was considered equivocal for regional nodal involvement, and, to avoid understaging, the post-surgical stage was assigned as INSS 2B. The renal pelvis and large renal vessels showed no tumor invasion, and renal parenchyma away from the main tumor mass was free of malignancy. Immunohistochemistry showed strong expression of neuronal markers (synaptophysin, chromogranin, and CD56) and negativity for WT1, desmin, and myogenin, excluding Wilms tumor and rhabdomyosarcoma. Array-based copy-number analysis of the primary tumor was available three weeks after surgery and showed multiple segmental chromosomal aberrations (SCAs), including deletions at 1p and 3p, in the absence of MYCN amplification and pathogenic ALK alterations, placing the tumor within an unfavorable SCA genomic category. Based on baseline imaging (prior to surgery), no image-defined risk factors (IDRFs) were identified; the tumor was therefore classified as International Neuroblastoma Risk Group (INRG) stage L1 [8,9]. Following nephrectomy/adrenalectomy and nodal sampling, the post-surgical stage was International Neuroblastoma Staging System (INSS) stage 2B due to regional lymph node involvement [10]. Bone marrow evaluation and an initial whole-body 123I-MIBG scan showed no metastatic disease apart from a small MIBG-avid focus in the operative bed corresponding to surgical clips, interpreted as minimal local residual disease.

### 3.3. Management and Outcome

Following complete macroscopic resection and an uncomplicated postoperative course, with no evidence of gross residual tumor or metastases, the tumor met criteria for low-risk disease (INSS stage 1), without indications for adjuvant chemotherapy. Although array-based copy-number profiling required approximately three weeks to become available, earlier access to these genomic results would not have changed the initial indication for chemotherapy, because treatment escalation is not recommended for a completely resected stage 1. The multidisciplinary team initially chose close observation, consistent with protocols that permit surveillance for selected infants with localized or completely resected neuroblastoma, but with a more intensive follow-up schedule due to genomic findings. 

At around 3 months of age, surveillance ultrasound showed a new heterogeneous mass in the left renal bed and several tiny echogenic foci in the liver. MRI confirmed suspicion of local recurrence, although assessment was hampered by postoperative changes. A repeat whole-body MIBG scan demonstrated enlargement of the operative-bed focus and multiple new MIBG-avid lesions in the posterior mediastinum, paravertebral region, and skeleton (skull base, orbit, ribs, long bones), consistent with early disseminated relapse. At relapse, the mIBG disease burden by SIOPEN scoring was 21. Bone marrow evaluation with automatic immunofluorescence plus FISH (AIPF) at relapse showed positive micrometastatic disease (68 cells per 7.4 million analyzed cells).

After imaging suggested progression, the patient received one cycle of carboplatin–etoposide according to an intermediate-risk neuroblastoma regimen. Following confirmation of disseminated relapse on MIBG scanning, the patient was reclassified as high risk, and systemic therapy was escalated to an intensive COJEC-based high-risk protocol, including vincristine, cisplatin, carboplatin, etoposide, and cyclophosphamide. Treatment was complicated by febrile neutropenia, Clostridioides difficile colitis, Staphylococcus aureus skin infection, and cisplatin-induced nephrotoxicity in the solitary kidney, requiring intensive supportive care. After induction chemotherapy, MRI showed approximately 80% regression of paravertebral masses and near-complete resolution of chest wall and intramuscular lesions. A small residual abdominal mass in the left upper quadrant was resected, and histology confirmed neuroblastoma, now stroma-rich with prominent treatment-related changes. Histology confirmed residual neuroblastoma with a marked treatment effect. Genomic analysis of the residual tumor revealed a normal copy-number profile, without detectable SCAs or MYCN/ALK amplification. The normal copy-number profile observed in the post-therapy residual specimen should be interpreted cautiously. Potential explanations include low viable tumor cellularity in a stroma-rich, treatment-affected sample; sampling heterogeneity (analysis of a less aberrant component); and/or therapy-induced clonal selection with preferential eradication of the SCA-bearing clone detected at diagnosis.

The patient developed CTCAE grade 2 nephrotoxicity, with a peak serum creatinine of 0.69 mg/dL (reference range 0.20–0.40 mg/dL), nadir estimated glomerular filtration rate (eGFR) of 43 mL/min/1.73 m^2^, urea 54 mg/dL (reference range 11–43 mg/dL), and cystatin C 1.87 mg/L (reference range 0.59–1.04 mg/L). The cumulative administered doses were cisplatin 240 mg/m^2^, carboplatin 1500 mg/m^2^, and cyclophosphamide 2100 mg/m^2^, which were associated with acute kidney injury.

In view of cisplatin-related nephrotoxicity, consolidation was switched to TVD (topotecan, vincristine, and doxorubicin) rather than further platinum-based therapy. Two cycles were administered with acceptable tolerance and stabilization of renal function. After a substantial radiological response to induction chemotherapy, peripheral blood stem cells were collected by apheresis. High-dose chemotherapy with autologous stem cell rescue and radiotherapy were deferred, balancing potential benefit against late-effect risks in a young child with a solitary kidney who had achieved near-complete remission. Post-consolidation MRI of the abdomen and spine and chest imaging showed near-complete resolution of all known lesions and no new disease. Bone marrow examinations were negative, and tumor markers had normalized. Clinically, the child was well, with appropriate growth and development and no neurological deficits. Renal function of the remaining kidney was stable but requires long-term monitoring. Given the excellent response, lack of MYCN amplification, evolving tumor biology, and concern about cumulative toxicity, the multidisciplinary team opted for close surveillance rather than immediate high-dose therapy. A schedule of follow-up visits, with imaging and laboratory tests, was established. At the last follow-up at the age of 14 months after completion of chemotherapy, the patient remains in complete remission; follow-up from completion of therapy is 6 months. Late-effect surveillance includes normal complete remission, with normal renal function and blood pressure. Longer-term monitoring is ongoing, particularly for renal function in the solitary kidney, hearing, and cardiac function, as the late sequelae cannot yet be fully assessed and warrant ongoing surveillance.

## 4. Discussion

Neuroblastoma is the most common extracranial solid malignancy of childhood and usually arises from the adrenal medulla or paraspinal sympathetic chain. Adrenal neuroblastoma typically presents as a suprarenal mass displacing the kidney, but it may infiltrate renal parenchyma or, rarely, arise from ectopic intrarenal sympathetic tissue [11]. In such situations, neuroblastoma can closely mimic a primary renal tumor, and a number of cases have initially been misdiagnosed as Wilms tumor [12,13,14]. This case illustrates how typical age-based differential diagnosis and imaging findings can bias diagnosis in neonatal abdominal masses. In the first months of life, CMN is the dominant renal tumor, and Wilms tumor is very rare. In older children, Wilms tumor becomes the prevailing renal malignancy. By contrast, neuroblastoma is common overall but usually presents as an adrenal or paraspinal tumor rather than a renal one.

Classically, adrenal neuroblastoma tends to be extrarenal, displacing the kidney, encasing vessels, and often showing calcifications, whereas Wilms tumor arises from and enlarges the kidney. In small infants, however, tumors are often small, the planes between the adrenal and kidney are narrow, calcifications may be absent, and vessel encasement is subtle [11,15]. Under these conditions, neuroblastoma can appear indistinguishable from a primary renal tumor on ultrasound and MRI, reminding us of the longstanding recommendation to evaluate urinary catecholamines in all suprarenal masses. Urinary catecholamine metabolites were not obtained pre-operatively, as imaging favored nephroblastomatosis/early Wilms tumor, and the lesion was managed as a presumed primary renal tumor—we acknowledge this as a diagnostic limitation. In light of this case, we would now recommend routine urinary catecholamine screening (VMA/HVA) in neonates with an upper-pole “renal” mass or any lesion at the adrenal–renal interface, particularly when the adrenal gland is not clearly separable on imaging.

This case highlights that radiological impressions, in particular in very young infants, should be regarded as provisional and that definitive classification of an ambiguous renal/adrenal mass frequently requires histopathology, immunohistochemistry, and, increasingly, molecular diagnostics.

A second challenge involved risk classification and initial treatment intensity. Based on age (<18 months), localized disease, and absence of MYCN amplification, this infant met criteria for low-risk neuroblastoma in many traditional algorithms, where surgery alone with observation is acceptable for selected patients [8,9]. Standard low-risk infant neuroblastoma management is typically risk-adapted: INRG stage L1 disease is often treated with upfront surgery and observation, whereas INRG L2 (localized tumors with image-defined risk factors) and an SCA profile generally prompt neoadjuvant chemotherapy followed by surgery, with additional postoperative treatment in selected cases. In metastatic disease (INRG stage M), first-line therapy commonly consists of up to six induction cycles (four cycles of VP–carboplatin and two cycles of CADO) [16,17].

In this case, array-based profiling showed multiple segmental chromosomal aberrations, including 1p and 3p loss. SCAs are recognized adverse prognostic markers even in non-MYCN-amplified neuroblastoma and are incorporated into INRG-based risk stratification [18]. Infants with localized disease but SCA profiles have a higher risk of relapse than those with tumors showing only whole-chromosome changes. The very early and disseminated relapse in this patient is consistent with the recognized association between unfavorable SCA profiles and increased relapse risk, but causal inferences from a single case should be avoided. The initial decision prioritized avoidance of overtreatment in a neonate with apparently localized, resected, non–MYCN-amplified disease; however, the subsequently confirmed unfavorable SCA profile (1p/3p loss) indicated higher relapse risk. Notably, the array-based copy-number result was available only three weeks after surgery, i.e., after the initial post-operative decision for observation had already been made. Earlier access to an unfavorable SCA profile might have lowered the threshold for initiating systemic therapy or intensifying surveillance despite an apparently localized presentation; however, in practice, such genomic profiling required tumor tissue and was therefore dependent on surgical sampling in this case.

In hindsight, this case raises the possibility that some infants with otherwise favorable clinical features but unfavorable genomic risk markers may benefit from a lower threshold for systemic therapy. Given the individual clinical course and relapse pattern, we elected to treat our patient using an intensified high-risk-oriented COJEC regimen, which is frequently used in relapsed settings and, in the case of unfavorable response or genetic profile evolution, offers the option to escalate to high-dose therapy, radiotherapy, and immunotherapy if required [19,20]. However, in this neonate with a solitary kidney and clinically significant platinum-associated nephrotoxicity, we prioritized organ preservation and reduction in late effects once a near-complete remission was achieved. Accordingly, we did not proceed with high-dose chemotherapy or radiotherapy due to age- and toxicity-related concerns.

Finally, it is worth noting that an increased risk of neuroblastoma has been reported in association with certain congenital anomalies, particularly involving the gastrointestinal tract and cardiovascular system [21]. However, a specific and reproducible link between isolated cleft lip/palate and neuroblastoma has not been established [22]. Germline predisposition testing was not performed in this case; we acknowledge this as a limitation and note that germline testing may be considered in selected patients, depending on clinical features and family history. Other limitations include the single-patient nature of the report, thus limiting generalizability, and missing marker studies at diagnosis.

## 5. Conclusions

This case underlines the importance of multidisciplinary decision-making, integrating clinical factors, genomic risk, treatment response, and organ function to balance cure against late effects. While an unfavorable SCA profile coincided with an aggressive early relapse pattern in this patient, the treatment implications of SCAs in neonates with apparently localized disease warrant validation in larger and prospective cohorts.

## Data Availability

The original contributions presented in this study are included in the article. Further inquiries can be directed to the corresponding author.
